# Identification and Validation of a Gene Signature for Lower-Grade Gliomas Based on Pyroptosis-Related Genes to Predict Survival and Response to Immune Checkpoint Inhibitors

**DOI:** 10.1155/2022/8704127

**Published:** 2022-04-30

**Authors:** Guichuan Lai, Kangjie Li, Jielian Deng, Hui Liu, Biao Xie, Xiaoni Zhong

**Affiliations:** Department of Epidemiology and Health Statistics, School of Public Health and Management, Chongqing Medical University, Yixue Road, Chongqing 400016, China

## Abstract

Pyroptosis plays a critical role in the immune response to immune checkpoint inhibitors (ICIs) by mediating the tumor immune microenvironment. However, the impact of pyroptosis-related biomarkers on the prognosis and efficacy of ICIs in patients with lower-grade gliomas (LGGs) is unclear. An unsupervised clustering analysis identified pyroptosis-related subtypes (PRSs) based on the expression profile of 47 pyroptosis-related genes in The Cancer Genome Atlas-LGG cohort. A PRS gene signature was established using univariate Cox regression, random survival forest, least absolute shrinkage and selection operator, and stepwise multivariable Cox regression analyses. The predictive power of this signature was validated in the Chinese Glioma Genome Atlas database. We also investigated the differences between high- and low-risk groups in terms of the tumor immune microenvironment, tumor mutation, and response to target therapy and ICIs. The PRS gene signature comprised eight PRS genes, which independently predicted the prognosis of LGG patients. High-risk patients had a worse overall survival than did the low-risk patients. The high-risk group also displayed a higher proportion of M1 macrophages and CD8^+^ T cells and higher immune scores, tumor mutational burden, immunophenoscore, IMmuno-PREdictive Score, MHC I association immune score, and T cell-inflamed gene expression profile scores, but lower suppressor cells scores, and were more suitable candidates for ICI treatment. Higher risk scores were more frequent in patients who responded to ICIs using data from the ImmuCellAI website. The presently established PRS gene signature can be validated in melanoma patients treated with real ICI treatment. This signature is valuable in predicting prognosis and ICI treatment of LGG patients, pending further prospective verification.

## 1. Introduction

Lower-grade gliomas (LGGs) in the brain arise from neuroepithelial heterogeneous tissue derived from glial cells of the central nervous system [1]. The World Health Organization classified grade I and II gliomas as “low-grade” based on histopathological characteristics [2]. However, grades II and III tumors were recently defined as “lower-grade glioma,” considering that isocitrate dehydrogenase (IDH) mutations appear in most grade II and III gliomas but rarely in grade IV tumors [3]. The main treatment strategies for LGGs include surgery, radiotherapy, chemotherapy, and immunotherapy. They all improve survival to a certain extent. However, some patients may not respond to these therapies because of tumor heterogeneity [4]. Furthermore, the efficacy of radiotherapy and chemotherapy is poor for LGG patients due to the lack of a continuous response [3]. Immunotherapy has recently been reported as a promising therapeutic approach for various types of cancers [5]. Immune checkpoint inhibitors (ICIs) to programmed cell death protein 1 (PD-1), programmed death-ligand 1 (PD-L1), and cytotoxic T lymphocyte-associated antigen-4 (CTLA-4) can activate antitumor immunity and mediate cancer recession [6]. However, due to the presence or absence of immunogenicity, not all patients respond well to ICIs [5]. The tumor mutation burden (TMB), expression of immune checkpoints (ICs), and tumor-infiltrating lymphocytes are considered biomarkers of the efficacy of ICI treatment and have been used to choose patients who could potentially benefit from subsequent therapy with ICIs [7–9]. However, it is inappropriate to choose immunotherapy schemes based on a single criterion when ignoring the intrinsic relationship of these biomarkers. It has been suggested that TMB should be integrated with other ICI-related biomarkers [10]. Therefore, it is necessary to develop a signature associated with these ICI-related biomarkers to predict the response of patients to ICIs.

Many studies have focused on the prediction of patient prognosis and efficacies of treatments that include chemotherapy, radiotherapy, and immunotherapy, by establishing immune-related, tumor microenvironment- (TME-) related, and tumor mutation-related gene signatures related to prognosis [11–13]. However, few studies have explored the relationship between pyroptosis-related biomarkers and the prediction of prognosis and efficacy of LGG treatments. As an inflammatory and programmed mode of cell death, pyroptosis plays a critical role in the immune response to ICIs [14]. How pyroptosis activates antitumor immunity remains unclear, although the association between pyroptosis and TME has attracted research attention for many years. Prior studies have provided some explanatory factors. Pyroptosis can inhibit tumor growth by transforming immune “cold” tumors into “hot” tumors, resulting in the infiltration of various immune cells [15, 16]. Inflammation caused by pyroptosis may promote the release of inflammatory mediators, such as interleukin (IL)-1 and IL-18, which construct an inflammatory microenvironment suitable for tumor development [15, 17]. On the other hand, pyroptosis can stimulate antitumor immunity and enhance tumor cell growth because of the heterogeneity of tumors and the complexity of the tumor immune microenvironment [14]. Pyroptosis alone is unable to induce effective antitumor inhibition. Pyroptosis combined with ICI treatment can effectively reduce “cold” tumors [18, 19]. Therefore, combining ICI treatment and pyroptosis is a prospective strategy to improve the prognosis of LGG patients and their response to ICIs.

In this study, we divided LGG patients into different clusters through consensus unsupervised clustering analysis based on 47 pyroptosis-related genes (PRGs). Pyroptosis-related subtype (PRS) genes were used to construct a PRS gene signature associated with the prognosis of LGG patients depending on the random survival forest (RSF) and least absolute shrinkage and selection operator (LASSO) algorithms. Patients were divided into high- and low-risk groups according to their median risk score. The ability of this signature to predict the prognosis and relationship between the PRS gene signature and TME, tumor mutation, and efficacy of ICIs were assessed in the two risk groups. The aim was to fully combine pyroptosis and antitumor immune response based on the bioinformatics data.

## 2. Materials and Methods

### 2.1. Data Collection and Processing

Transcriptomic data and clinical information of LGG patients were obtained from UCSC Xena, The Cancer Genome Atlas (TCGA) (https://xenabrowser.net/), and Chinese Glioma Genome Atlas (CCGA) (http://www.cgga.org.cn/) databases. The gene expression profile from TCGA (version: 07-19-2019) was measured experimentally using the Illumina platform. This dataset showed the gene-level transcription estimates, which were log2 (*x* + 1) transformed count and fragments per kilobase of exon per million mapped fragments (FPKM). The log_2_ (count+1) and log_2_ (FPKM+1) were then converted into count and FPKM, respectively. FPKM data was finally transformed into transcript per million (TPM) according to the formula: TPM_*i*_ = FPKM_*i*_ ∗ 1000000/(FPKM_0_ + ⋯ + FPKM_*m*_), where *i* represented gene *i* and *m* represented the total number of all genes. The count data was used for differential expression analysis and TPM data was used for other analyses. We used the GRCh38.104 from the Ensembl (http://Asia.ensembl.org/) database to annotate these genes. Gene expression profiles in CGGA-325 (version: 05-06-2020) and CGGA-693 (version: 05-06-2020) were obtained using Illumina HiSeq 2000 or 2500 and the Illumina HiSeq platform, respectively. Next, the two sets of gene expression data were corrected in batches and integrated by the “limma” [20] and “sva” packages [21]. Only primary, grade II, and grade III patients with complete survival information were included in this study. Detailed information of LGG patients was shown in Table 1. The clinical features included age, gender, tumor grade, follow-up time, and survival status.

### 2.2. Construction of PRS

Forty-seven PRGs were obtained from a previously published article (Supplementary Table 1) [22]. “*K*-means” consensus unsupervised clustering analysis based on Euclidean and Ward's linkage was employed to classify patients into distinct molecular subtypes according to mRNA expression data of the 47 PRGs by R package “ConsensusClusterPlus” in TCGA. The optimal *k* was determined by the proportion of ambiguous clustering [23], cumulative distribution function (CDF), and relative change in area under the CDF curve. One thousand repetitions were performed to ensure classification stability.

### 2.3. Characteristics of PRS

The R packages “survival” and “survminer” were used to analyze overall survival (OS) difference and differential expression analysis was performed using the R package “edgeR” among different subtypes [24]. Differentially expressed PRS genes were selected with |log_2_Foldchange| >2 and a false discovery rate <0.05. Gene ontology (GO) enrichment consisted of cellular components, biological property, and molecular function. Kyoto Encyclopedia of Genes and Genomes (KEGG) pathway analysis was performed with the “BH” method and adjusted *p* value <0.05 to investigate the biological function of these PRS genes through the R package “clusterprofiler” [25].

### 2.4. Identification and Validation of PRS Gene Signature

First, we identified prognostic PRS genes by univariate Cox regression analysis with *p* < 0.05. These PRS genes relevant to the prognosis of LGG patients were introduced into RSF analysis using the R package “randomForestSRC” to rank the prognostic genes in accordance with the variable relative importance score (VRIS). A VRIS <0 indicated a reduction of the prediction accuracy, while VRIS >0 suggested an improvement in prediction accuracy. Variables with VRIS >0 were selected for the LASSO algorithm, which was used to limit overfitting and obtain optimized prognostic PRS genes using the R package “glmnet.” The genes with nonzero regression coefficients selected from LASSO regression analysis were enrolled in the stepwise multivariate Cox regression analysis to construct a PRS gene signature to predict the prognosis of LGG patients and their response to ICIs. The risk score of each LGG patient was calculated through a pyroptosis-related risk model.

(1) $$\begin{array}{c} \text{Risk score}=\sum_{i=1}^{n}coef_{i}*\;\exp_{i}. \end{array}$$

The coef_*i*_ and exp_*i*_ defined the regression coefficient and expression of each gene, respectively. Patients in the TCGA and CGGA cohorts were divided into high- and low-risk groups using the median value of risk score as the cut-off value and subjected to Kaplan-Meier survival analysis. In addition, the time-independent receiver operating characteristics (ROC) curve was used to assess the predictive capability of this signature via the R package “time-ROC.”

### 2.5. Immunohistochemistry (IHC) and Prognosis of LGG Patients at the Level of Eight PRS Genes

IHC staining images of the PRS genes in LGG patients and normal brain tissues were obtained from the Human Protein Atlas (HPA) (http://www.proteinatlas.org/) database. The Gene Expression Profiling Interactive Analysis (GEPIA) (http://gepia.cancer-pku.cn/) database was used to further confirm the correlation of these PRS genes with prognosis in LGG patients.

### 2.6. Clinical Features and Presently Established Gene Signature

To determine whether the presently established gene signature can be an independent prognostic factor for LGG patients, risk scores and clinical information were incorporated into univariate and multivariate Cox regression analyses. Variables with *p* < 0.05 were statistically significant.

### 2.7. Comparison of Previous Pyroptosis-Related Signatures in LGG Patients

Four previously determined pyroptosis-related signatures associated with the prognosis of glioma patients were collected [26–29]. They were compared with the presently established signature in four external datasets: including CGGA, E-MATB-3892 (https://www.ebi.ac.uk/arrayexpress/), E-MTAB-2768 (https://www.ebi.ac.uk/arrayexpress/), and Rembrandt (http://www.cgga.org.cn/download_other.jsp). The AUC and Kaplan-Meier survival curves were used to compare the predictive ability.

### 2.8. Tumor Immune Microenvironment and Presently Established Gene Signature

The proportion of tumor-infiltrating immune cells was estimated by the “CIBERSORT” (https://cibersort.stanford.edu/) deconvolution algorithm characterizing the cell composition based on normalized gene expression profiles. The gene expression matrix of 22 immune cells was collected from the leucocyte signature matrix 22 [30]. One thousand permutations were run using the CIBERSORT algorithm. Samples with *p* < 0.05 were included in the tumor-infiltrating analysis. Considering the complexity of TME which consisted of immune and stromal cells, immune and stromal scores were calculated by the “ESTIMATE” algorithm, which is usually used to quantify the TME [31].

### 2.9. TMB and Presently Established PRS Gene Signature

Mutation profiling was acquired from TCGA by the “maftools” package [32]. TMB is an important indicator of the efficacy of ICIs, estimated as (total mutation/total covered bases) ×10^6^. We explored the association of some mutated biomarkers with our PRS gene signature. The distribution of mutated genes was visualized by the “maftools” package. TMB was divided into high- and low- TMB groups on the basis of the median values. Mutant genes that differed between these groups were used to compare with the corresponding wild-type genes regarding the prognosis of LGG patients.

### 2.10. Significance of PRS Gene Signature in Predicting Response to Targeted Therapy and ICIs

Immunophenoscore (IPS), MHC I association immune score (MIAS), T cell-inflamed gene expression profile (GEP) score, and IMmuno-PREdictive Score (IMPRES) were calculated by the IPS (https://github.com/icbi-lab/Immunophenogram) and MIAS (https://github.com/perwu/MIAS) R scripts, respectively. IPS is a method that evaluates a patient's relative probability to respond to ICIs based on some important components of tumor immunity, including major histocompatibility complex (MHC), checkpoints (CP), effector cells (EC), and suppressor cells (SC) [33]. IPS ranges from 0 to 10, with higher IPS indicating a higher response to ICI treatment. IMPRES ranges from 0 to 15, with higher IMPRES reflecting a higher relative probability of response to ICIs [34]. The MIAS and GEP methods were used to predict PD-1 blockade treatment; the high predictive value has been recently verified in melanoma patients [35, 36]. Another way to assess the prediction of ICIs was via the ImmuCellAI (http://bioinfo.life.hust.edu.cn/web/ImmuCellAI/) website, which was developed based on an ssGSEA algorithm [37]. To evaluate the prediction of our signature in a real immunotherapy cohort, we used 49 melanoma patients receiving ICI treatment from the GSE91061 (http://www.ncbi.nlm.nih.gov/geo/) dataset to further validate our findings. We used the “pRRophetic” package to evaluate the response of the LGG patients to lapatinib, an epidermal growth factor receptor inhibitor [38]. The IC50 value of each patient was calculated using Ridge's regression based on the Genomics of Drug Sensitivity in Cancer database (http://www.cancerrxgene.org/).

### 2.11. Statistical Analysis

All statistical analyses were performed using R software. Stepwise multivariable Cox regression analysis was used to construct the PRS signature. OS between high- and low-risk groups was compared using Kaplan-Meier survival curves with log-rank tests. AUC was used to identify the predictive capacity of time-independent ROC curves. The Wilcoxon test was applied to compare the proportion of tumor-infiltrating immune cells, immune and stromal scores, TMB, ICs, IPS, and others. The *p* value was two-sided, and *p* < 0.05 was considered statistically significant.

## 3. Results

### 3.1. Characteristics of Patients

**Figure 1:**
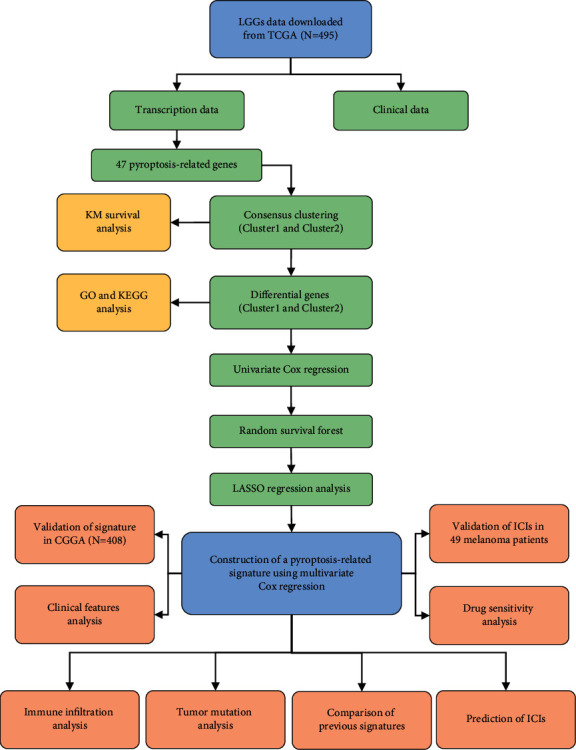
The experimental flow chart in this study.

A total of 495 primary, grade II, and grade III LGG patients with complete survival data in TCGA were included as the training set in this study. In addition, 408 LGG patients from the CGGA-325 and CGGA-693 cohorts with the same selected criteria comprised the test set for external validation. The experimental flow chart was shown in Figure 1.

### 3.2. Identification and Characteristics of PRS in LGG Patients

**Figure 2:**
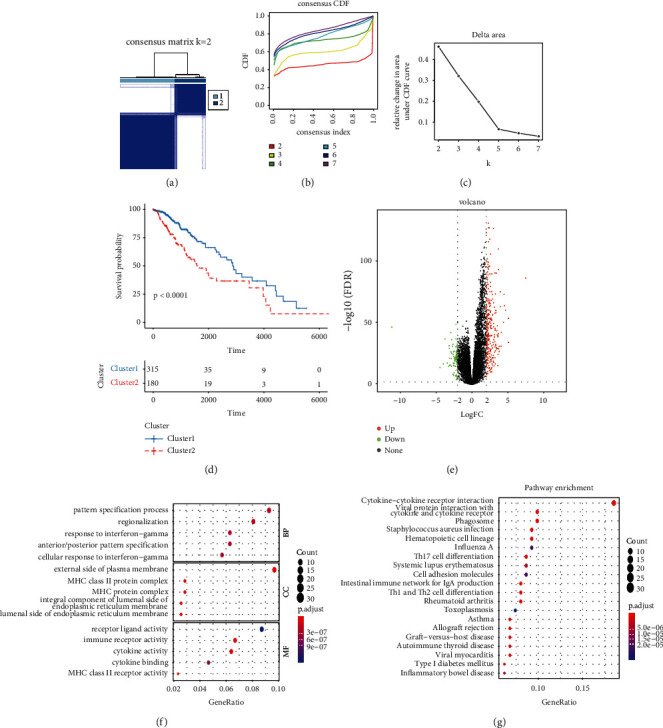
Identification and characteristics of PRS. (a) Consensus matrix heatmap of two subtypes (*k* = 2). (b) The correlation between CDF and consensus index under consensus CDF curve when *k* = 2–7. (c) The relative change in area under the CDF curve when *k* = 2–7. (d) Kaplan-Meier survival analysis of OS between Cluster1 and Cluster2. (e) The volcano plot of differentially expressed PRS genes. (f) GO enrichment analysis of differentially expressed PRS genes. (g) KEGG pathway enrichment analysis of differentially expressed PRS genes. BP, biology process; CC, cellular component; MF, molecular function.

Consensus unsupervised clustering analysis based on the expression profile of 47 PRGs was used to identify the potential molecular subtypes of the LGG patients. The highest intragroup and lowest intergroup correlations appeared when TCGA-LGG patients were accurately classified into two subtypes (Figures 2(a)–2(c); Supplementary Table 2). Kaplan-Meier survival curves showed the patients with cluster1 had a significantly longer OS than those with cluster2 (log-rank test, *p* < 0.0001; Figure 2(d)). Consensus unsupervised clustering analysis successfully divided 408 patients from the CGGA dataset into two subtypes based on 47 PRGs (Supplementary Figure 1(a-c)). Survival was better for the patients in cluster2 than for those in cluster1 (log-rank test, *p* = 0.00018; Supplementary Figure 1(d)). In the TCGA-LGG cohort, 377 differentially expressed PRS genes between the clusters that were identified comprised 289 upregulated and 88 downregulated genes (Figure 2(e); Supplementary Table 3). Finally, GO enrichment and KEGG pathway analyses of 377 differentially expressed PRS genes further explored the potential biological function between the different subtypes. These 377 differentially expressed PRS genes were mainly enriched in biological functions associated with immunity, including response to interferon-gamma (INF-*γ*), MHC class II protein complex, MHC protein complex, MHC class II receptor activity, immune receptor activity, cytokine activity, cytokine-cytokine receptor interaction, Th17 cell differentiation, Th1 and Th2 cell differentiation, and cell adhesion molecules (Figures 2(f) and 2(g); Supplementary Table 4, 5).

### 3.3. Construction and Validation of PRS Gene Signature

**Figure 3:**
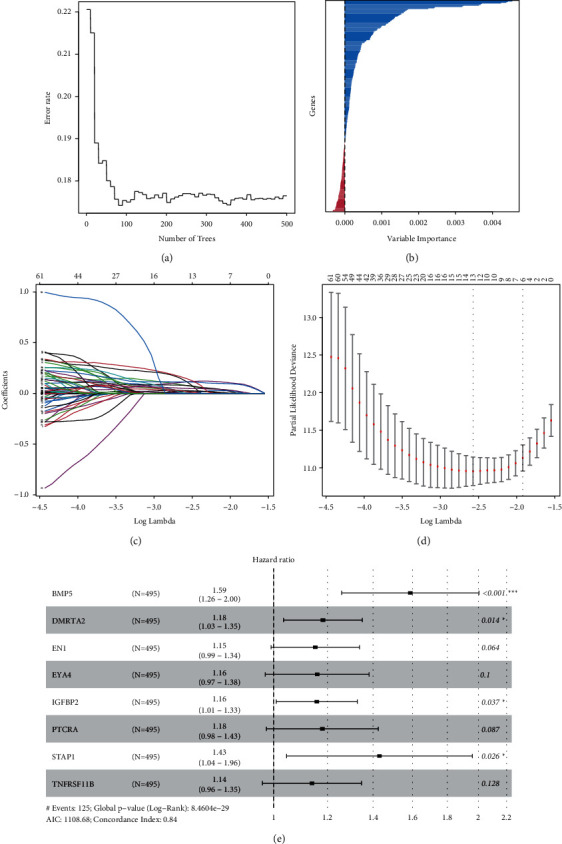
The construction of PRS gene signature. (a) The change of error rate with the number of trees in the RSF model. (b) The relative importance score distribution of PRS genes in the RSF model. (c) LASSO coefficient profiles of the 204 prognostic PRS genes. (d) Partial likelihood deviance of genes revealed by LASSO. (e) Forest plot of each gene in eight-gene PRS signature after stepwise multivariate Cox regression analysis.

**Figure 4:**
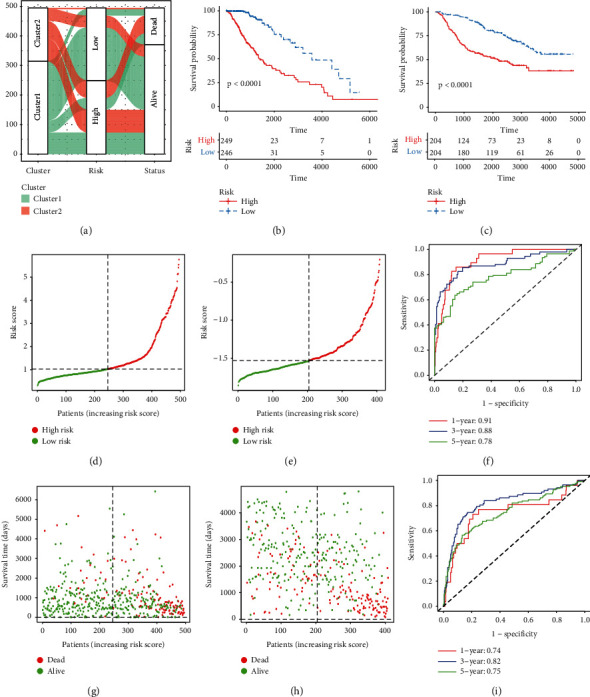
The validation of an eight-gene PRS signature. (a) Alluvial diagram of different subtypes with different risk scores and survival outcomes. (b) Kaplan-Meier survival curves of OS between the high-risk group and low-risk group in TCGA. (c) Kaplan-Meier survival curves of OS between the high-risk group and low-risk group in CGGA. (d) The distribution of risk scores in TCGA. (e) The distribution of risk scores in CGGA. (f) The time-dependent ROC curves of 1-, 3-, and 5-year in TCGA. (g) The relationship between survival status, survival time, and risk score in TCGA. (h) The relationship between survival status, survival time, and risk score in CGGA. (i) The time-dependent ROC curves of 1-, 3-, and 5-year in CGGA.

Univariate Cox regression among 495 primary LGG patients identified 305 differentially expressed PRS genes associated with OS (*P* < 0.05; Supplementary Table 6). Subsequently, 204 prognostic PRS genes with VRIS >0 via the RSF algorithm (Figures 3(a) and 3(b); Supplementary Table 7) were chosen for the LASSO algorithm (Figures 3(c) and 3(d); Supplementary Table 8) analysis. Thirteen PRS genes were assessed by multivariate Cox regression analysis. Finally, an eight-gene PRS gene signature was constructed (Figures 3(e)). Riskscore = (BMP5 ∗ 0.46289) + (DMRTA2 ∗ 0.16702) + (EN1 ∗ 0.14118) + (EYA4 ∗ 0.14763) + (IGFBP2 ∗ 0.14614) + (PTCRA ∗ 0.16520) + (STAP1 ∗ 0.35830) + (TNFRSF11B ∗ 0.13016). The distribution of patients in the two subtypes was displayed in Figure 4(a). Time-independent ROC and Kaplan-Meier survival curves were used to evaluate the predictive capacity of our PRS signature. Higher risk scores were correlated with worse OS (log-rank test, *p* < 0.0001; Figures 4(b) and 4(c)). The AUC of 1-, 3-, and 5-year OS was 0.91, 0.88, and 0.78, respectively, in TCGA-LGG cohort (Figure 4(f)) and 0.74, 0.82, and 0.75, respectively, in CGGA-LGG cohort (Figure 4(i)) of patients. Risk curve and survival distribution maps of LGG patients were used to further assess the discriminatory power in TCGA (Figures 4(d) and 4(g)) and CGGA (Figures 4(e) and 4(h)). Our PRS gene signature displayed high distinctive and predictive capacities.

### 3.4. Verification of IHC and Prognosis of LGG Patients at the Level of PRS Genes

BMP5, IGFBP2, and PTCRA are not available on the HPA website. Thus, we compared the other five genes of normal tissue and tumor tissue that are available on this website. The staining of DMRTA2 was medium in tumor tissue and undetectable in normal tissue (Supplementary Figure 2(a)). EYA4 was weakly positive in tumor tissue and negative in normal tissue (Supplementary Figure 2(b)). EN1, STAP1, and TNFRSF11B were negative in both normal and tumor tissues (Supplementary Figure 2(c-e)). LGG patients with higher expression of these PRS genes tended to have a worse OS in this study (log-rank test, *p* < 0.05; Supplementary Figure 3).

### 3.5. PRS Gene Signature Was an Independent Prognostic Factor for LGG Patients

**Figure 5:**
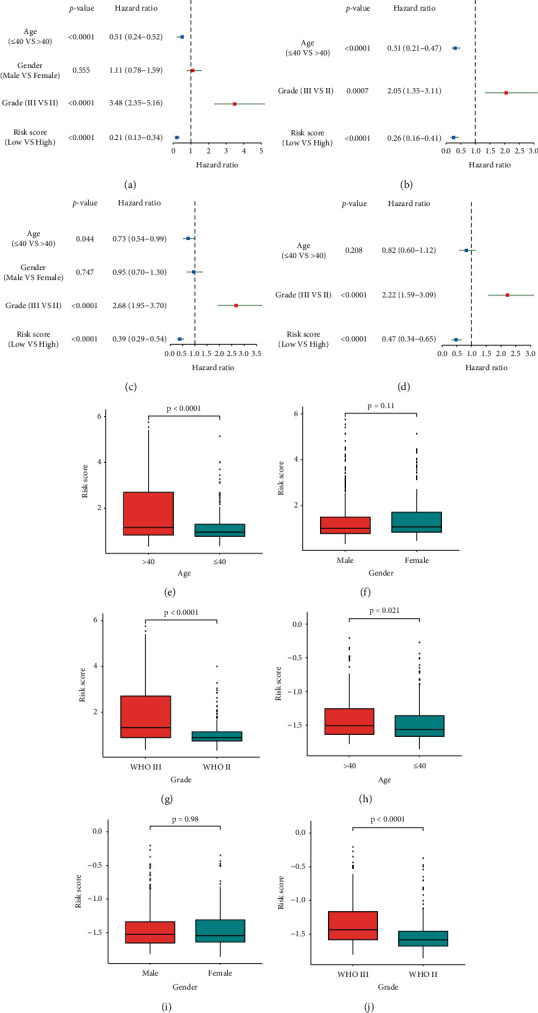
The eight-gene PRS signature and clinical features. (a) The forest plot of univariate Cox regression analysis in TCGA. (b) The forest plot of multivariate Cox regression analysis in TCGA. (c) The forest plot of univariate Cox regression analysis in CGGA. (d) The forest plot of multivariate Cox regression analysis in CGGA. (e-g) Risk score in LGG patients with different age, gender, and grade groups in TCGA. (h-j) Risk score in LGG patients with different age, gender, and grade groups in CGGA.

Tumor grade and risk score were related to prognosis in both univariate and multivariate Cox regression analyses (*p* < 0.001; Figures 5(a)–5(d)). After adjusting for confounding factors, our PRS gene signature was an independent predictive factor for LGGs. High tumor grade and high-risk score were risk factors for prognosis, but there was no statistical significance in other clinical features, such as age and gender of LGG patients. Patients with high age and tumor grade tended to have high-risk scores (Wilcoxon test, *p* < 0.05; Figures 5(e), 5(g), 5(h), and 5(j)), but no statistical significance was found in gender group (Wilcoxon test, *p* < 0.05; Figures 5(f) and 5(i)).

### 3.6. Predictive Power of PRS Gene Signature Compared with Similar Signatures

The presently established gene signature and a prior signature statistically stratified patients into high- and low-risk categories in Kaplan-Meier survival analyses. The AUC of our signature was higher than those of other signatures in four datasets. The difference was most evident with the Rembrandt dataset (Supplementary Figures 4–9).

### 3.7. PRS Gene Signature and Tumor Immune Microenvironment

**Figure 6:**
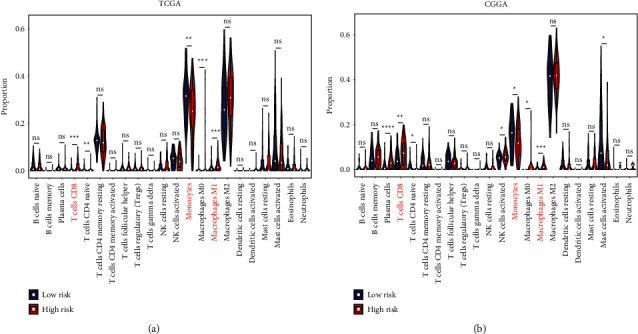
The relationship between tumor-infiltrating immune cells and PRS gene signature. (a) The proportion of tumor-infiltrating immune cells between the low-risk group and high-risk group in TCGA. (b) The proportion of tumor-infiltrating immune cells between the low-risk group and high-risk group in CGGA. Data in (a-b) were analyzed by Wilcoxon test; ns, no significance; ^∗^*p* < 0.05, ^∗∗^*p* < 0.01, and ^∗∗∗^*p* < 0.001.

**Figure 7:**
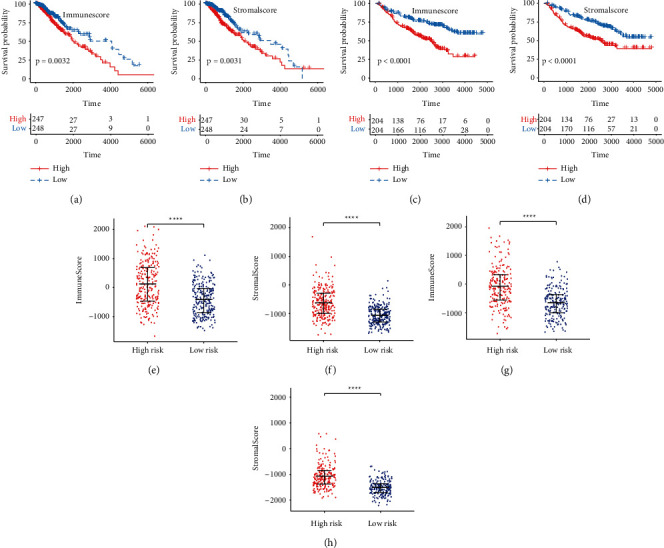
The relationship between TME and OS, and PRS gene signature. (a-b) Kaplan-Meier survival analysis of immune score and stromal score in TCGA. (c-d) Kaplan-Meier survival analysis of immune score and stromal score in CGGA. (e-f) The immune score and stromal score between the high-risk group and low-risk group in TCGA. (g-h) The immune score and stromal score between the high-risk group and low-risk group in CGGA. Data in (e-h) were analyzed by Wilcoxon test; ^∗^*p* < 0.05, ^∗∗^*p* < 0.01, ^∗∗∗^*p* < 0.001, and ^∗∗∗∗^*p* < 0.0001.

We calculated the proportion of 22 immune cells using the CIBERSORT algorithm to investigate the relationship between presently established gene signature and tumor immune microenvironment. In high-risk TCGA-LGG patients, a higher proportion of M0 and M1 macrophages, CD4 naïve T cells, and CD8 T cells, and a lower proportion of monocytes were found (Wilcoxon test, *p* < 0.01; Figure 6(a); Supplementary Table 9). The CIBERSORT algorithm was used to assess the gene expression of CGGA-LGG patients to ensure the stability of the immune infiltration results. High-risk CGGA-LGG patients displayed a higher proportion of M1 macrophages, plasma cells, and CD8 T cells, but a lower proportion of monocytes were observed (Wilcoxon test, *p* < 0.05; Figure 6(b); Supplementary Table 10). The combining results from the two cohorts demonstrated that LGG patients with high-risk scores had a higher proportion of M1 macrophages and CD8 T cells, but a lower proportion of monocytes, than those with low-risk scores. Finally, the ESTIMATE algorithm was applied to compare the immune and stromal scores between the high- and low-risk groups. Higher immune and stromal scores were found in the high-risk group compared with the low-risk group in both TCGA (Wilcoxon test, *p* < 0.0001; Figures 7(e) and 7(f)); Supplementary Table 11) and CGGA cohorts (Wilcoxon test, *p* < 0.0001; Figures 7(g) and 7(h)); Supplementary Table 12). Patients with lower immune and stromal scores had a significantly longer OS in both TCGA (log-rank test, *p* < 0.01; Figures 7(a) and 7(b)) and CGGA (log-rank test, *p* < 0.0001; Figures 7(c) and 7(d)) cohorts.

### 3.8. PRS Gene Signature and TMB

**Figure 8:**
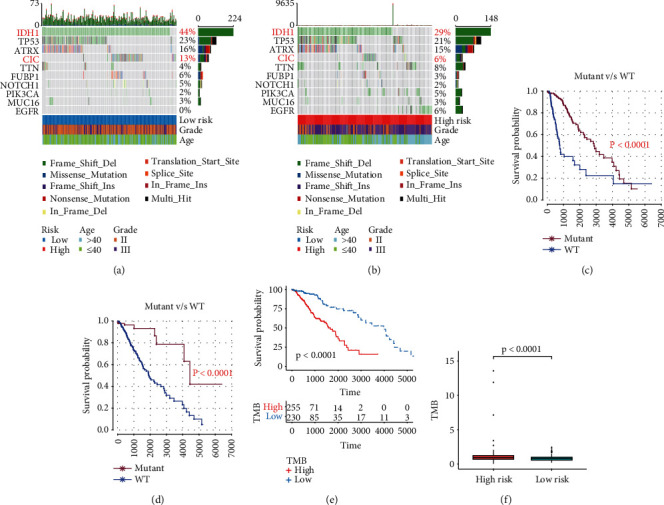
The relationship between tumor mutation and PRS gene signature. (a) Mutation profile of top 10 mutated genes in the low-risk group. (b) Mutation profile of top 10 mutated genes in the high-risk group. (c) Kaplan-Meier survival analysis between patients with wild-type IDH1 and mutant IDH1. (d) Kaplan-Meier survival analysis between patients with wild-type CIC and mutant CIC. (e) Kaplan-Meier survival analysis between the low-TMB group and high-TMB group. (f) The TMB between the low-risk group and high-risk group.

The top 10 most significantly mutated genes were IDH1, TP53, ATRX, CIC, TTN, FUBP1, NOTCH1, PIK3CA, MUC16, and EGFR in TCGA. Low-risk patients had a higher frequency of IDH1 (chi-square test; *p* < 0.001; Figures 8(a) and 8(b)) and CIC (chi-square test; *p* = 0.016; Figures 8(a) and 8(b)) mutation than high-risk patients. Patients with wild-type IDH1 and CIC had a worse OS than those with mutated IDH1 and CIC (*p* < 0.0001; Figures 8(c) and 8(d)). After calculating the TMB of each patient, higher TMB was related to a shorter OS (log-rank test, *p* < 0.0001; Figure 8(e)). TMB was obviously higher in the high-risk group compared to the low-risk group (Wilcoxon test, *p* < 0.0001; Figure 8(f); Supplementary Table 13).

### 3.9. Response of Patients to Target Therapy and ICIs

**Figure 9:**
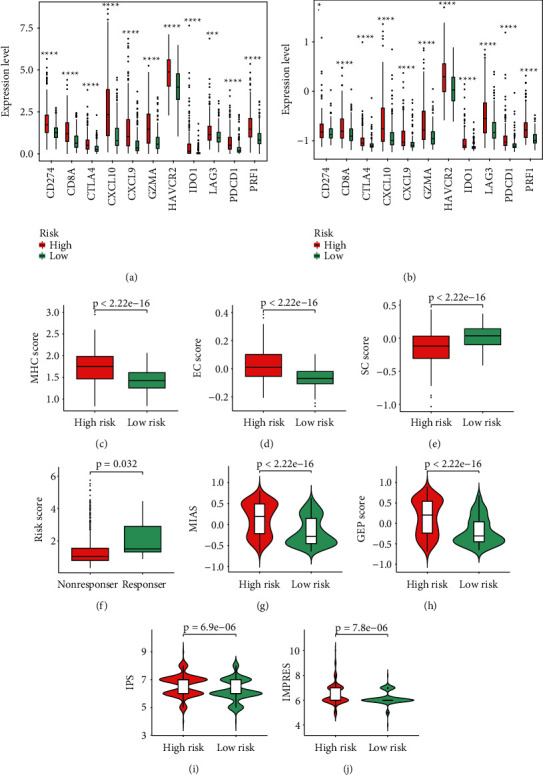
LGG patients' response to ICIs and PRS gene signature. (a) The expressions of 11 ICs between the low-risk group and high-risk group in TCGA. (b) The expressions of 11 ICs between the low-risk group and high-risk group in CGGA. (c-e) The MHC score, EC score, and SC score between the low-risk group and high-risk group. (f) Risk score in LGG patients with a different ICI response status. (g-j) The MIAS, GEP score, IPS, and IMPRES between the low-risk group and high-risk group. Data in (a-b) were analyzed by Wilcoxon test; ^∗^*p* < 0.05, ^∗∗^*p* < 0.01, ^∗∗∗^*p* < 0.001, and ^∗∗∗∗^*p* < 0.0001.

**Figure 10:**
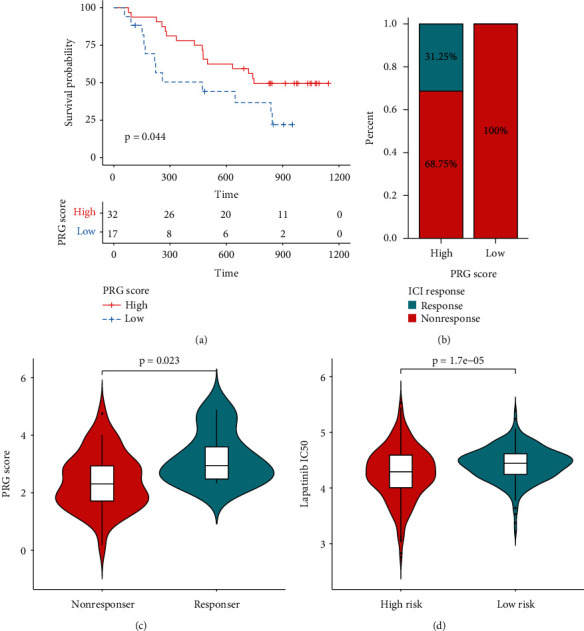
The role of PRS gene signature in the prediction of immunotherapeutic benefits and targeted therapy. (a) Kaplan-Meier survival curves for 49 melanoma patients with high and low PRG scores in GSE91061. (b) Rate of 49 melanoma patients' clinical responses to ICI treatment in high and low PRG scores in GSE91061. (c) PRG score in 49 melanoma patients with a different ICI response status in GSE91061. (d) Lapatinib IC50 value of LGG patients between the high-risk group and low-risk group.

High-risk patients displayed higher expression of ICs, including CD274, CD8A, CTLA4, CXCL10, CXCL9, GZMA, HAVCR2, IDO1, LAG3, PDCD1, and PRF1 in both TCGA (Wilcoxon test, *p* < 0.001, Figure 9(a)) and CGGA (Wilcoxon test, *p* < 0.05, Figure 9(b)) cohorts. These patients also displayed higher MHC scores, EC scores, IPS, MIAS, GEP scores, and IMPRES, but lower SC scores (Wilcoxon test, *p* < 0.0001; Figures 9(c), 9(e), 9(g), and 9(j); Supplementary Table 14). The collective findings indicated the higher sensitivity of high-risk patients to ICI treatment. The response status of LGG patients according to gene expression was determined through the ImmuCellAI website. Responders had higher risk scores than nonresponders (Wilcoxon test, *p* = 0.032; Figure 9(f); Supplementary Table 15). The presently established gene signature effectively predicted the prognosis of melanoma patients (log-rank test, *p* = 0.044; Figures 10(a) and 10(b)). These patients who responded to ICIs had higher PRG scores compared to nonresponders (Wilcoxon test, *p* = 0.023; Figure 10(c)). Higher estimated IC50 values of lapatinib were obtained in low-risk patients compared with high-risk patients, indicating the sensitivity of high-risk patients to this drug (Wilcoxon test, *p* < 0.0001; Figure 10(d); Supplementary Table 16). These results supported the fact that high-risk LGG patients had a higher relative probability of response to ICIs.

## 4. Discussion

Increasing evidence indicates that pyroptosis induced by inflammation influences the TME and directly or indirectly activates the immune response to tumors [15, 16]. However, a comprehensive tool needs to be developed with the realization of the “double-edged sword” nature of that pyroptosis, and that tumor growth is difficult to inhibit by pyroptosis alone [14, 15, 17]. Increasing research attention has focused on PRGs in the development of cancer. However, few studies have considered the pyroptosis-related status of patients and the association of pyroptosis with prognosis.

In this study, we used unsupervised clustering analysis to classify LGG patients into two subtypes (cluster1 and 2) based on 47 PRGs to assess the pyroptosis-related status. Compared to cluster2 patients, those in cluster1 had a better prognosis. The finding indicated that pyroptosis can influence the development of LGGs. Participation of differentially expressed PRS genes between the two clusters in various immune-related activities and mechanisms was revealed. These included response to IFN-*γ*, MHC class II protein complex, MHC protein complex, MHC protein complex, cytokine binding, cytokine-cytokine receptor interaction, and cell adhesion molecules. These findings indicated the association of pyroptosis with immune-related functions. IFN-*γ* can suppress tumors, increase MHC expression, enhance the function of tumor-infiltrating immune cells, and is involved in antigen presentation [39]. MHC is a genetic region that consists of MHC class I and II molecules. MHC-II has been associated with favorable outcomes in patients suffering from various solid cancers treated with immunotherapies [40, 41]. Adhesion molecules play vital roles in the function of the immune system and participate in every process of the antitumor response [42]. These findings highlight the potential value of PRS genes as an immunotherapy target.

In the present study, an eight-gene signature utilized to predict the prognosis and response to ICIs was constructed based on 47 PRS genes identified by RSF, LASSO, and multivariate Cox regression analyses. Of these eight PRS genes, three (BMP5, TNFRSF11B, and IGFBP2) are immune-related genes, and their association with the prognosis of cancer patients has been previously reported [43–45]. EN1, EYA4, IGFBP2, and PTCRA were also discovered as predictive biomarkers in the prognosis and treatments of LGG patients [46–49]. The expression of EN1 and EYA4 in LGGs was prevalent among some known tumor types. Higher expression of these two proteins has been correlated with shorter OS [46, 47]. High expression of IGFBP2 was detected in LGG tumor tissues compared with normal brain tissues. This expression was associated with a worse prognosis for LGG patients [48]. PTCRA was a biomarker associated with the prognosis of LGGs. Lower expression of PTCRA was related to longer OS [49]. These findings were all consistent with the results of our study.

In the present study, the PRS gene signature could precisely predict the OS of LGG patients in the training and validation cohorts. This gene signature was an independent predictor for the prognosis of LGGs in TCGA and CGGA cohorts when considering relevant clinical features, such as the tumor grade, age, and gender. Clinical variables with high-risk scores statistically tended to be risk factors for the prognosis, suggesting that the PRS gene signature can be a predictor for the prognosis and could be a substitute for prognosis. To investigate the prognostic mechanism of the signature and provide clues for the prediction of ICIs, we compared high- and low-risk groups in terms of the proportion of 22 immune cells, TME, gene mutation, TMB, ICs, and so on. Consistent with previous publications, infiltration of CD8 T cells and M1 macrophages was greater in the high-risk group compared with that in the low-risk group [50, 51]. Macrophages in the surrounding TME are usually termed tumor-associated macrophages and include M1 and M2 macrophages [52]. Unlike tumor-associated M2 macrophages, which led to an immunosuppressive TME and are actively involved in cancer metastasis, M1 macrophages are usually used as drug carriers for tumor therapy that directly kills tumor cells [52, 53]. Likewise, effector CD8^+^ T cells can inhibit tumor development and secrete several cytokines, including IFN-*γ* and IL-2 [54]. In terms of TME, high-risk patients displayed higher immune scores, indicating an enriched immune-related function in these patients. Feng et al. classified glioma patients into three groups (immunity-high, immunity-medium, and immunity-low) and demonstrated that the immunity-high patients had an unfavorable prognosis compared with the immunity-low patients [55]. The findings concerning the tumor immune microenvironment further support the idea that pyroptosis influences the development of LGGs by mediating this microenvironment.

Concerning tumor mutation, high-risk patients displayed a greater TMB than the low-risk patients, even though low-risk patients expressed more highly mutated genes (including the high frequency of mutant IDH1 and CIC). TMB was previously demonstrated to predict the outcome of ICI treatment. This is because higher TMB results in more neoantigens and enhanced T cell recognition and is clinically correlated with better outcomes of ICI therapy [56, 57]. Thus, high-risk patients may be more likely to respond to ICIs. Additionally, IDH1 mutation, as the main trait of LGGs, was characterized as low tumor mutational load, PD-1^+^ T cells, or PD-L1 expression [58]. Lin et al. described the higher expression of CD274, CTLA4, HAVCR2, PDCD1, and PDCD1LG2 in the CIC wild-type group of LGG patients [59]. Importantly, the TIDE scores in the CIC wild-type group were significantly lower than the scores in the CIC-mutant group [59]. Based on these findings, we can conclude that LGG patients in IDH1 wild-type and CIC wild-type groups were more likely to respond to ICI treatment, indicating that LGG patients with high-risk scores were more likely to respond to ICI treatment.

With an increased understanding of tumor immunology, immunotherapy has provided a new direction for tumor treatment. In the present study, we chose 11 ICs, including PDCD1, CD274, and CTLA-4, as ICI biomarkers and demonstrated higher levels of ICs in the high-risk group. Tumor growth is favored by upregulations of ICs in the TME [60]. Although the overexpression of ICs suppresses antitumor T cell responses, some studies have shown a strong positive correlation between IC ligand expression and response to IC blockade [61, 62]. For example, in one study, the risk of death was decreased by 34% in PD-L1 positive patients and by 20% in PD-L1 negative patients upon PD-1 or PD-L1 blockade treatment [61]. Patients with higher PD-L1 expression usually benefit more from anti-PD1 treatment [62]. Furthermore, the upregulation of these ICs in an inflamed tumor may initiate a contrary feedback mechanism that produces an active immune environment, which leads to an improved prognosis [63]. Except for these prevalent indicators for the prediction of ICIs, we evaluated the response to ICIs using some promising methods based on some genes associated with immunotherapy. We discovered that the IPS, IMPRES, MIAS, and the GEP, MHC, and EC scores were positively associated with risk scores. However, this association was not apparent for SC scores. Moreover, patients who responded to ICI treatments displayed high-risk scores compared with nonresponders using the ImmuCellAI website tool. Most importantly, our signature effectively predicted the efficacy of ICIs in melanoma patients from a real immunotherapy cohort.

These findings provide more evidence that high-risk LGG patients are more suitable for ICI treatment. However, there are several limitations to this study. The study was conducted based on retrospective data from public databases. However, we used various datasets to confirm the stability of these results. We assessed the likelihood of response of LGG patients to ICIs by some simulated values since we had limited access to actual immunotherapy data. We are trying to verify this PRS gene signature in melanoma patients from a real immunotherapy cohort. Finally, further experimental studies in vivo and in vitro are needed to confirm our results in the future.

## 5. Conclusion

We constructed an eight-gene PRS signature to predict LGG patients' prognosis and response to ICIs. High-risk scores were associated with a poor OS but were correlated with a high relative probability of response to ICIs. We believe our research can help optimize treatment plans and may be beneficial for improving the prognosis of LGG patients.

## Figures and Tables

**Table 1:** Clinical information of patients included in this study.

Variables	Group	TCGA (495)	CGGA (408)
Survival status	Alive	370	244
	Dead	125	164

Survival time (median time/days)	—	671	1927

Age	≤40	242	214
	>40	253	193
	Unknown	—	1

Gender	Male	274	236
	Female	221	172

Tumor grade	II	238	220
	III	256	188
	Unknown	1	—

## Data Availability

The results shown here are in whole or part based upon data generated by TCGA (https://xenabrowser.net/), GEO (http://www.ncbi.nlm.nih.gov/geo/), CGGA (http://www.cgga.org.cn/), Ensembl (http://Asia.ensembl.org/), HPA (http://www.proteinatlas.org/), GEPIA (http://gepia.cancer-pku.cn/), ArrayExpress (https://www.ebi.ac.uk/arrayexpress/), CIBERSORT (https://cibersort.stanford.edu/) databases, and ImmuCellAI (http://bioinfo.life.hust.edu.cn/web/ImmuCellAI/) website.

## Supplementary Materials

Supplementary Materials Supplementary 1. Supplementary Figure 1: validation of PRS in CGGA. Supplementary 2. Supplementary Figure 2: validation of five PRS genes at the protein level via IHC database. Supplementary 3. Supplementary Figure 3: validation of eight PRS genes regarding LGG patients' OS via GEPIA. Supplementary 4. Supplementary Figure 4: AUC comparison from five pyroptosis-related signatures. Supplementary 5. Supplementary Figure 5: Kaplan-Meier survival analysis using our signature. Supplementary 6. Supplementary Figure 6: Kaplan-Meier survival analysis using Chao B (2022). Supplementary 7. Supplementary Figure 7: Kaplan-Meier survival analysis using Zhang M (2021). Supplementary 8. Supplementary Figure 8: Kaplan-Meier survival analysis using Zhang Y (2021). Supplementary 9. Supplementary Figure 9: Kaplan-Meier survival analysis using Zheng J (2022). Supplementary 10. Supplementary Table 1: 47 pyroptosis-related genes. Supplementary 11. Supplementary Table 2: the result of consensus unsupervised clustering analysis in TCGA. Supplementary 12. Supplementary Table 3: 377 differentially expressed PRS genes. Supplementary 13. Supplementary Table 4: the result of GO enrichment analysis. Supplementary 14. Supplementary Table 5: the result of KEGG pathway analysis. Supplementary 15. Supplementary Table 6: the result of univariate Cox regression analysis. Supplementary 16. Supplementary Table 7: 204 genes with VRIS >0 in RSF. Supplementary 17. Supplementary Table 8: 13 genes with nonzero regression coefficients in LASSO. Supplementary 18. Supplementary Table 9: tumor-infiltrating immune cells in TCGA. Supplementary 19. Supplementary Table 10: tumor-infiltrating immune cells in CGGA. Supplementary 20. Supplementary Table 11: tumor microenvironment analysis by “ESTIMATE” in TCGA. Supplementary 21. Supplementary Table 12: tumor microenvironment analysis by “ESTIMATE” in CGGA. Supplementary 22. Supplementary Table 13: the result of TMB. Supplementary 23. Supplementary Table 14: the results of IPS, MIAS, GEP, and IMPRES. Supplementary 24. Supplementary Table 15: the result of ImmuCellAI. Supplementary 25. Supplementary Table 16: the result of drug sensitivity analysis.
