# Supplementary material for: Identification and Validation of a Gene Signature for Lower-Grade Gliomas Based on Pyroptosis-Related Genes to Predict Survival and Response to Immune Checkpoint Inhibitors
Source: J Healthc Eng. 2022 Apr 30;2022:8704127. doi: 10.1155/2022/8704127 (PMC9078805; doi:10.1155/2022/8704127)
Supplement: Supplementary Materials — Supplementary 1. Supplementary Figure 1: validation of PRS in CGGA. Supplementary 2. Supplementary Figure 2: validation of five PRS genes at the protein level via IHC database. Supplementary 3. Supplementary Figure 3: validation of eight PRS genes regarding LGG patients' OS via GEPIA. Supplementary 4. Supplementary Figure 4: AUC comparison from five pyroptosis-related signatures. Supplementary 5. Supplementary Figure 5: Kaplan-Meier survival analysis using our signature. Supplementary 6. Supplementary Figure 6: Kaplan-Meier survival analysis using Chao B (2022). Supplementary 7. Supplementary Figure 7: Kaplan-Meier survival analysis using Zhang M (2021). Supplementary 8. Supplementary Figure 8: Kaplan-Meier survival analysis using Zhang Y (2021). Supplementary 9. Supplementary Figure 9: Kaplan-Meier survival analysis using Zheng J (2022). Supplementary 10. Supplementary Table 1: 47 pyroptosis-related genes. Supplementary 11. Supplementary Table 2: the result of consensus unsupervised clustering analysis in TCGA. Supplementary 12. Supplementary Table 3: 377 differentially expressed PRS genes. Supplementary 13. Supplementary Table 4: the result of GO enrichment analysis. Supplementary 14. Supplementary Table 5: the result of KEGG pathway analysis. Supplementary 15. Supplementary Table 6: the result of univariate Cox regression analysis. Supplementary 16. Supplementary Table 7: 204 genes with VRIS >0 in RSF. Supplementary 17. Supplementary Table 8: 13 genes with nonzero regression coefficients in LASSO. Supplementary 18. Supplementary Table 9: tumor-infiltrating immune cells in TCGA. Supplementary 19. Supplementary Table 10: tumor-infiltrating immune cells in CGGA. Supplementary 20. Supplementary Table 11: tumor microenvironment analysis by “ESTIMATE” in TCGA. Supplementary 21. Supplementary Table 12: tumor microenvironment analysis by “ESTIMATE” in CGGA. Supplementary 22. Supplementary Table 13: the result of TMB. Supplementary 23. Supplementary Table 14: the results [file 8704127.f1.zip › 8704127.f1/Supplementary Figure1-9.pdf]

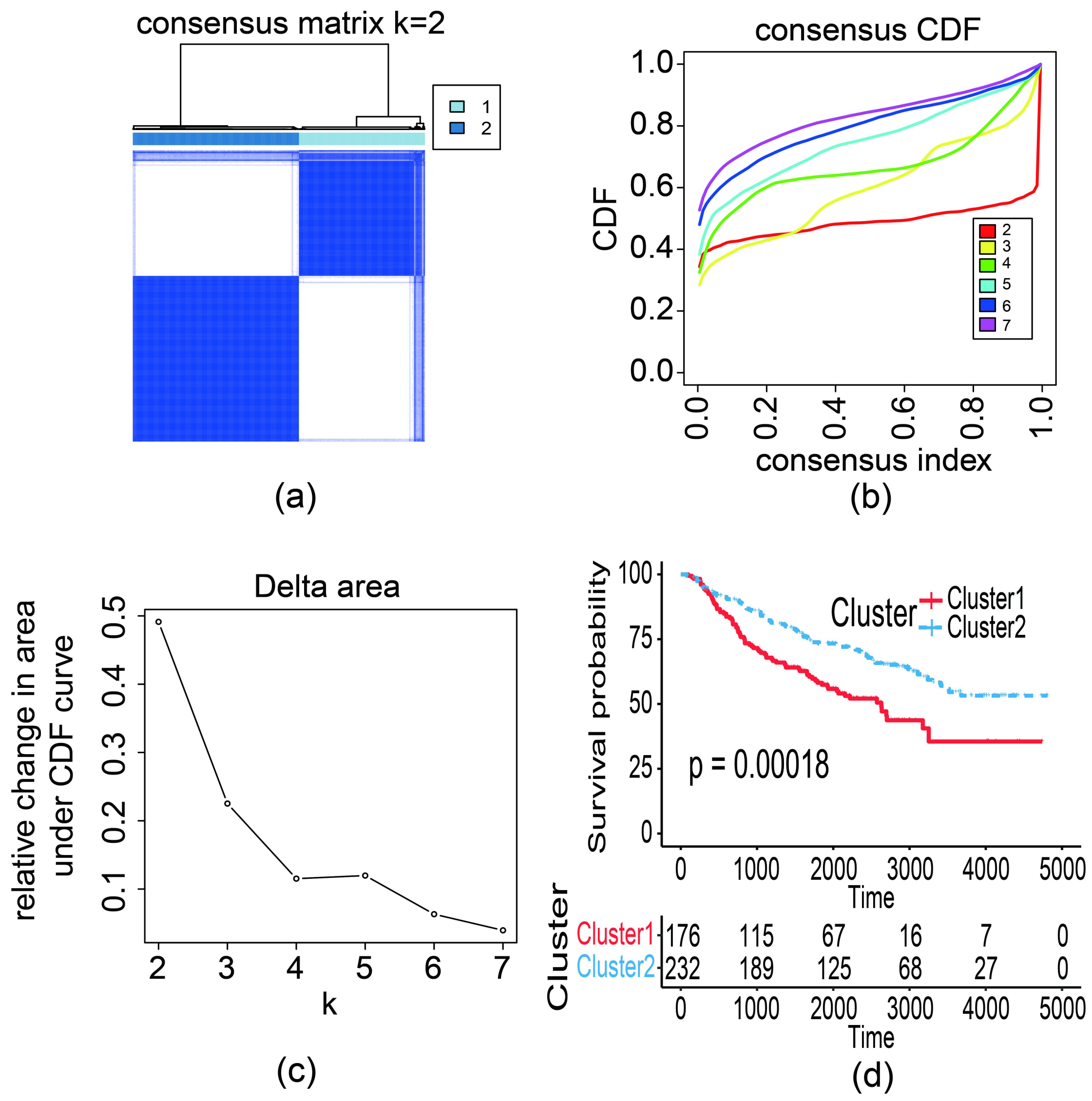

SUPPLEMENTARY FIGURE 1: Validation of PRS in CGGA. (a) Consensus matrix heatmap of two subtypes (k=2). (b) The correlation between CDF and consensus index under consensus CDF curve when k= 2-7. (c) The relative change in area under CDF curve when k=2-7. (d) Kaplan-Meier survival analysis of OS between Cluster1 and Cluster2.

DMRTA2

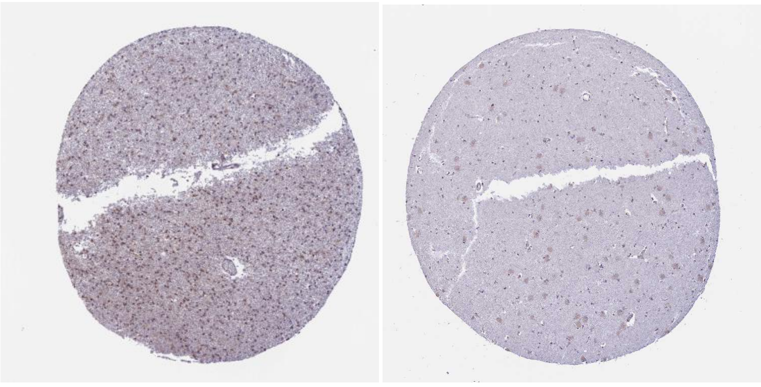

**HPA043098**	**HPA043098**
LGG tumor	Normal tissue
Staining: Medium	Staining: Not detected
Intensity: Moderate	Intensity: Week
Quantity: 75%-25%	Quantity: <25%

(a)

EYA4

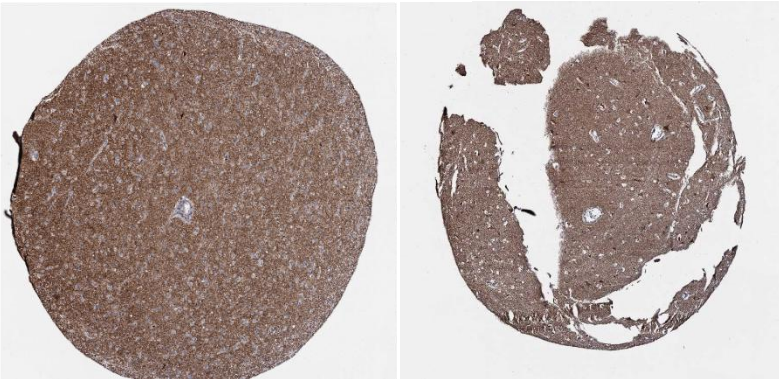

**HPA038772**	**HPA038772**
LGG tumor	Normal tissue
Staining: Not detected	Staining: Not detected
Intensity: week	Intensity: Negative
Quantity: <25%	Quantity: None

(b)

EN1

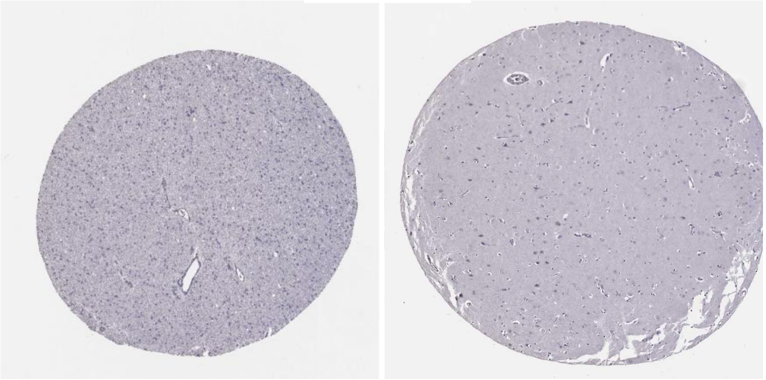

**HPA073141**	**HPA073141**
LGG tumor	Normal tissue
Staining: Not detected	Staining: Not detected
Intensity: Negative	Intensity: Negative
Quantity: None	Quantity: None

(c)

STAP1

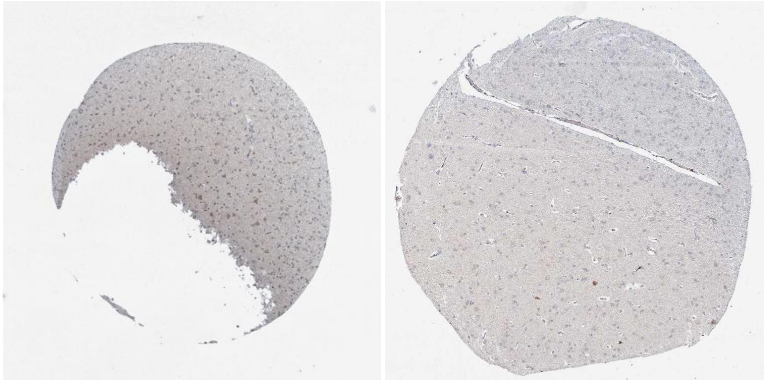

HPA038529
LGG tumor
Staining: Not detected
Intensity: Negative
Quantity: None

HPA038529
Normal tissue
Staining: Not detected
Intensity: Negative
Quantity: None

(d)

TNFRSF11B

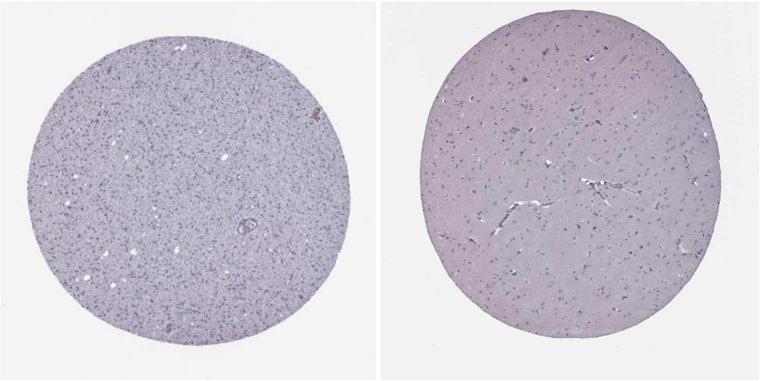

HPA034939
LGG tumor
Staining: Not detected
Intensity: Negative
Quantity: None

HPA034939
Normal tissue
Staining: Not detected
Intensity: Negative
Quantity: None

(e)

SUPPLEMENTARY FIGURE 2: Validation of five PRS genes at the protein level via IHC database. (a) Expression of DMRTA2 in LGG and normal tissues. (b) Expression of EYA4 in LGG and normal tissues. (c) Expression of EN1 in LGG and normal tissues. (d) Expression of STAP1 in LGG and normal tissues. (e) Expression of TNFRSF11B in LGG and normal tissues.

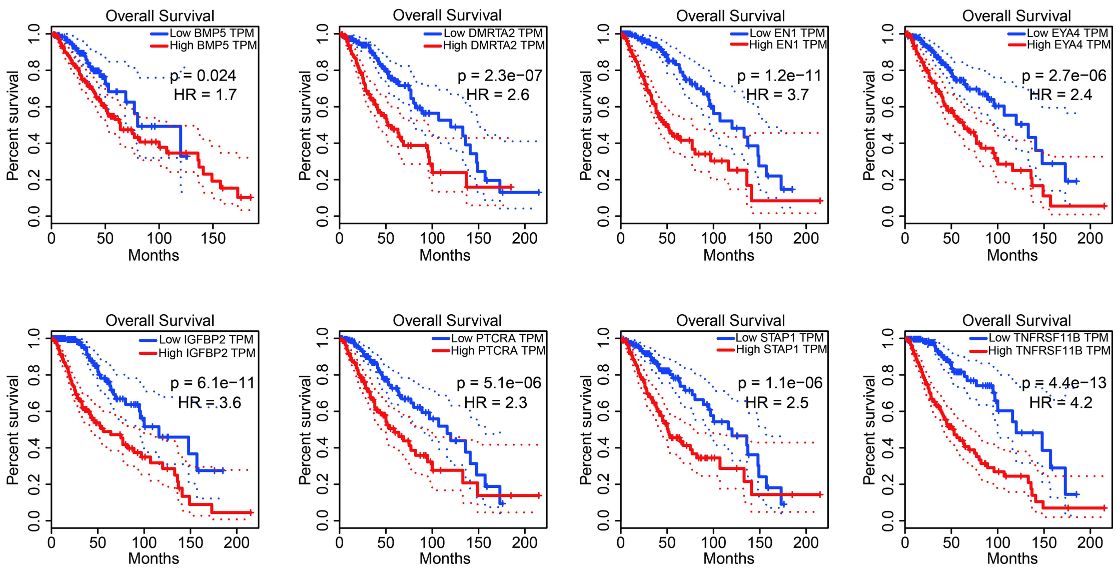

SUPPLEMENTARY FIGURE 3: Validation of eight PRS genes regarding LGG patients' OS via GEPIA website.

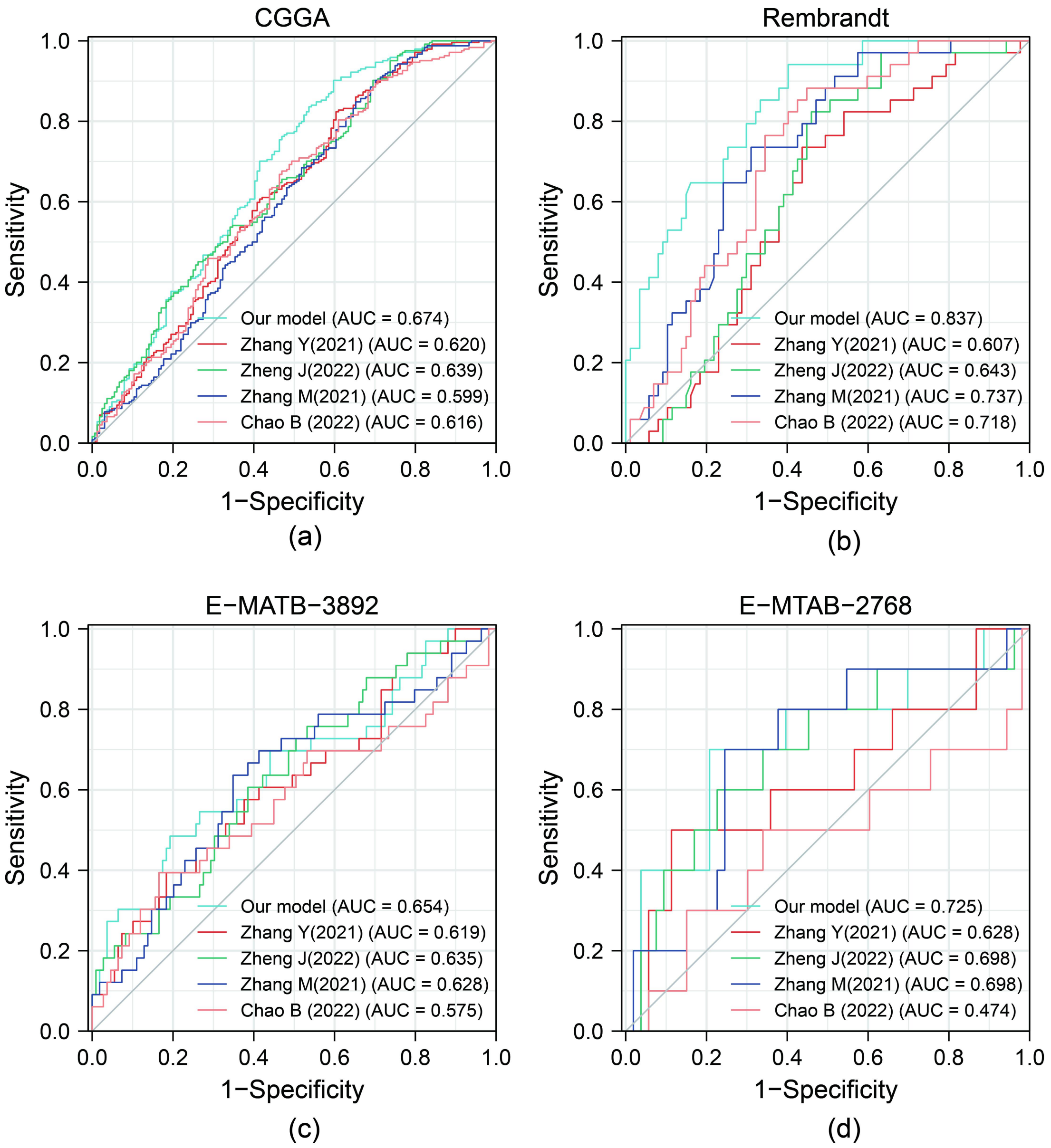

SUPPLEMENTARY FIGURE 4: AUC Comparison from five pyroptosis-related signatures in four external datasets. (a) Comparison of AUC in CGGA. (b) Comparison of AUC in Rembrandt. (c) Comparison of AUC in E-MATB-3892. (d) Comparison of AUC in E-MATB-2768.

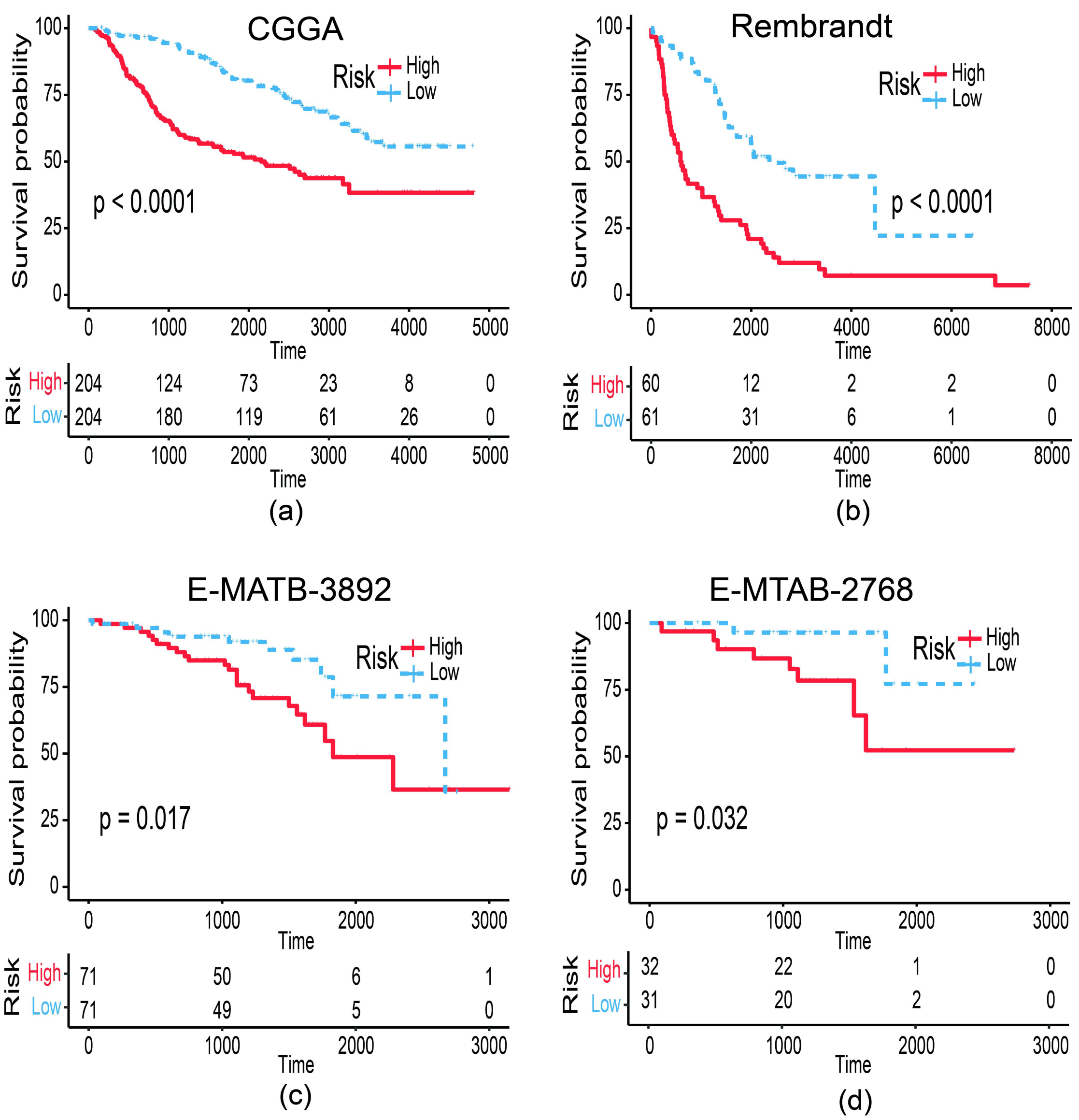

SUPPLEMENTARY FIGURE 5: Kaplan-Meier survival curves of OS between high-risk group and low-risk group using our signature in four external datasets. (a) Kaplan-Meier survival curves of OS between high-risk group and low-risk group in CGGA. (b) Kaplan-Meier survival curves of OS between high-risk group and low-risk group in Rembrandt. (c) Kaplan-Meier survival curves of OS between high-risk group and low-risk group in E-MATB-3892. (d) Kaplan-Meier survival curves of OS between high-risk group and low-risk group in E-MATB-2768.

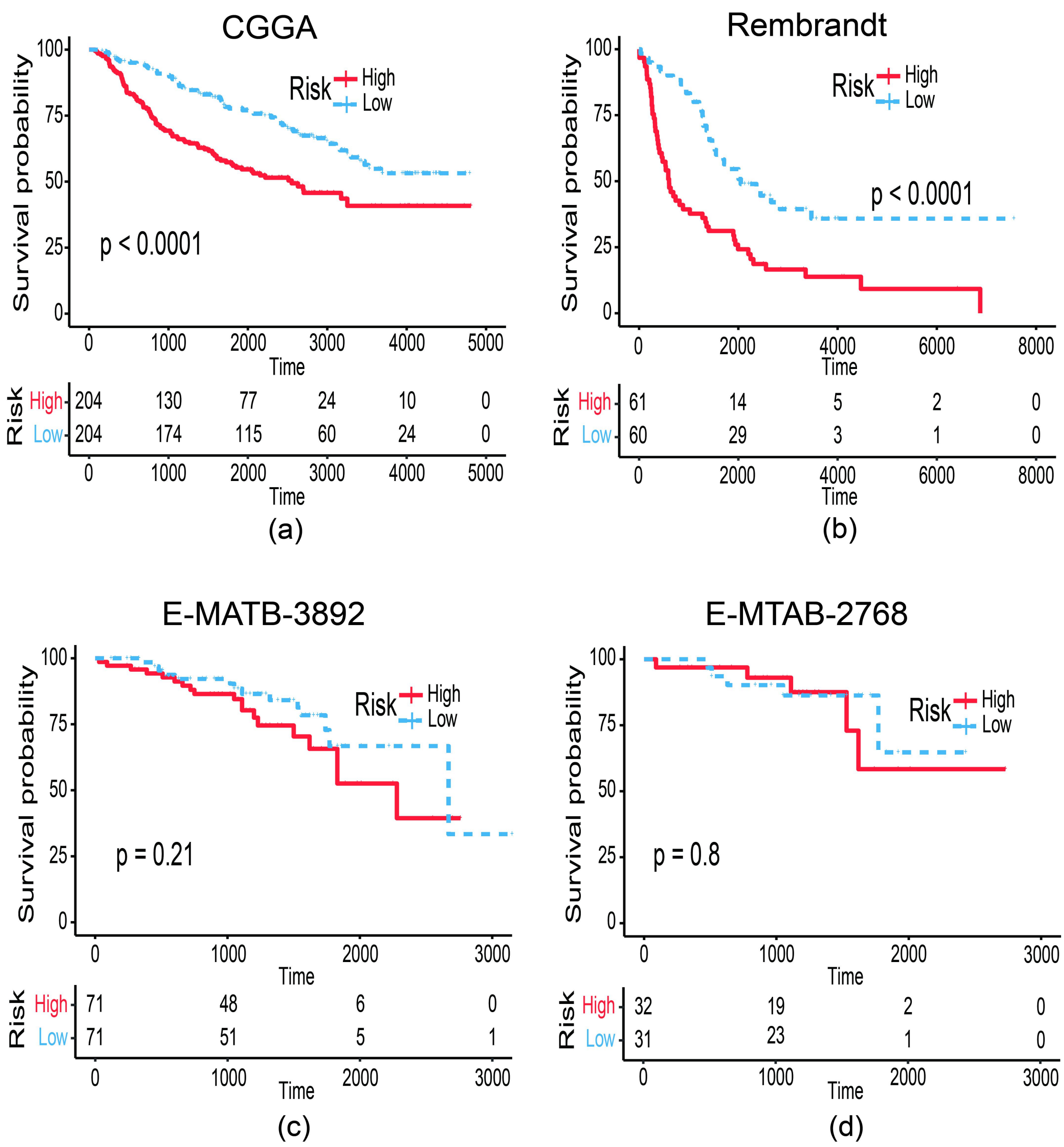

SUPPLEMENTARY FIGURE 6: Kaplan-Meier survival curves of OS between high-risk group and low-risk group using Chao B (2022) in four external datasets. (a) Kaplan-Meier survival curves of OS between high-risk group and low-risk group in CGGA. (b) Kaplan-Meier survival curves of OS between high-risk group and low-risk group in Rembrandt. (c) Kaplan-Meier survival curves of OS between high-risk group and low-risk group in E-MATB-3892. (d) Kaplan-Meier survival curves of OS between high-risk group and low-risk group in E-MATB-2768.

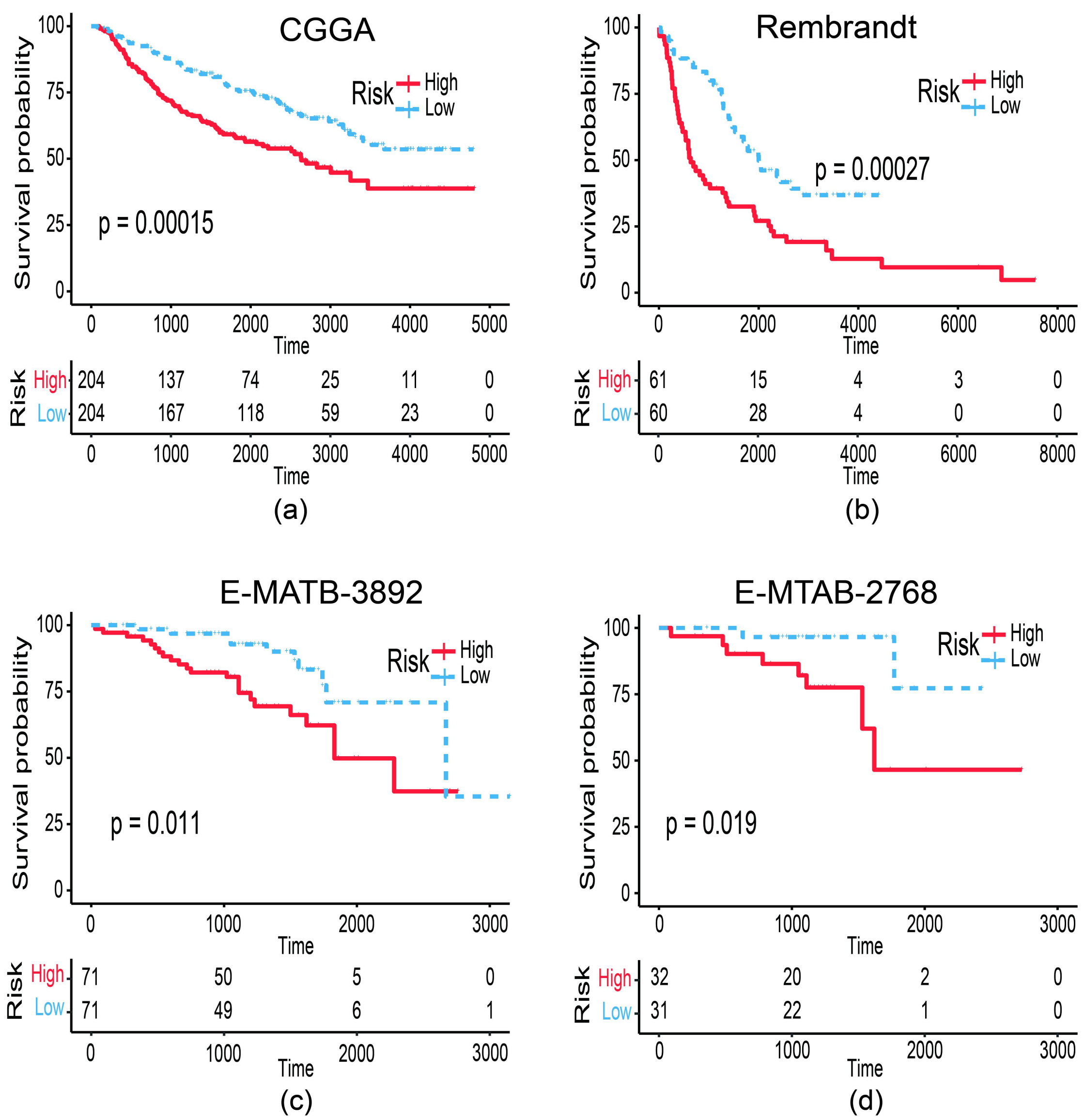

SUPPLEMENTARY FIGURE 7: Kaplan-Meier survival curves of OS between high-risk group and low-risk group using Zhang M (2021) in four external datasets. (a) Kaplan-Meier survival curves of OS between high-risk group and low-risk group in CGGA. (b) Kaplan-Meier survival curves of OS between high-risk group and low-risk group in Rembrandt. (c) Kaplan-Meier survival curves of OS between high-risk group and low-risk group in E-MATB-3892. (d) Kaplan-Meier survival curves of OS between high-risk group and low-risk group in E-MATB-2768.

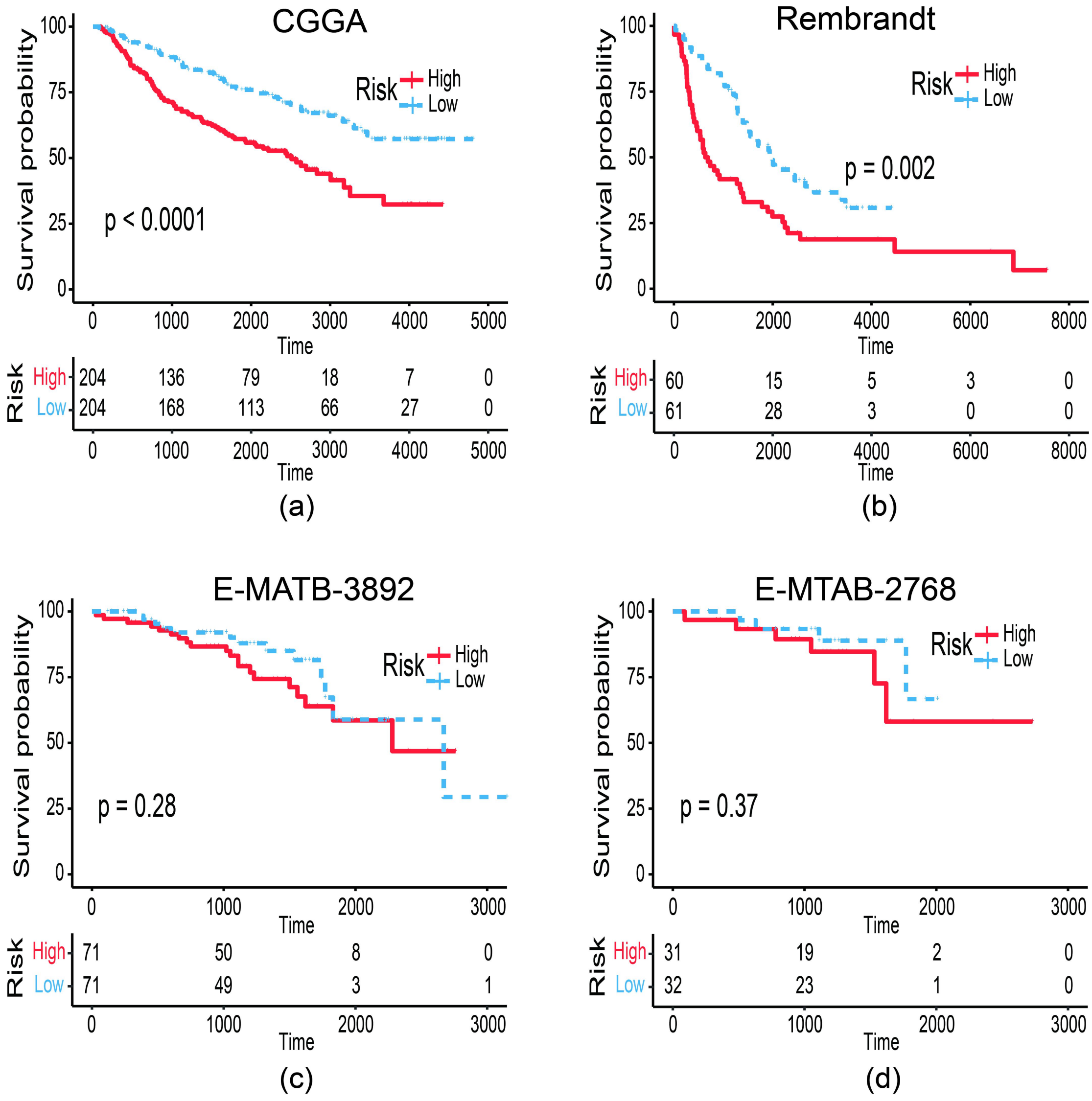

SUPPLEMENTARY FIGURE 8: Kaplan-Meier survival curves of OS between high-risk group and low-risk group using Zhang Y (2021) in four external datasets. (a) Kaplan-Meier survival curves of OS between high-risk group and low-risk group in CGGA. (b) Kaplan-Meier survival curves of OS between high-risk group and low-risk group in Rembrandt. (c) Kaplan-Meier survival curves of OS between high-risk group and low-risk group in E-MATB-3892. (d) Kaplan-Meier survival curves of OS between high-risk group and low-risk group in E-MATB-2768.

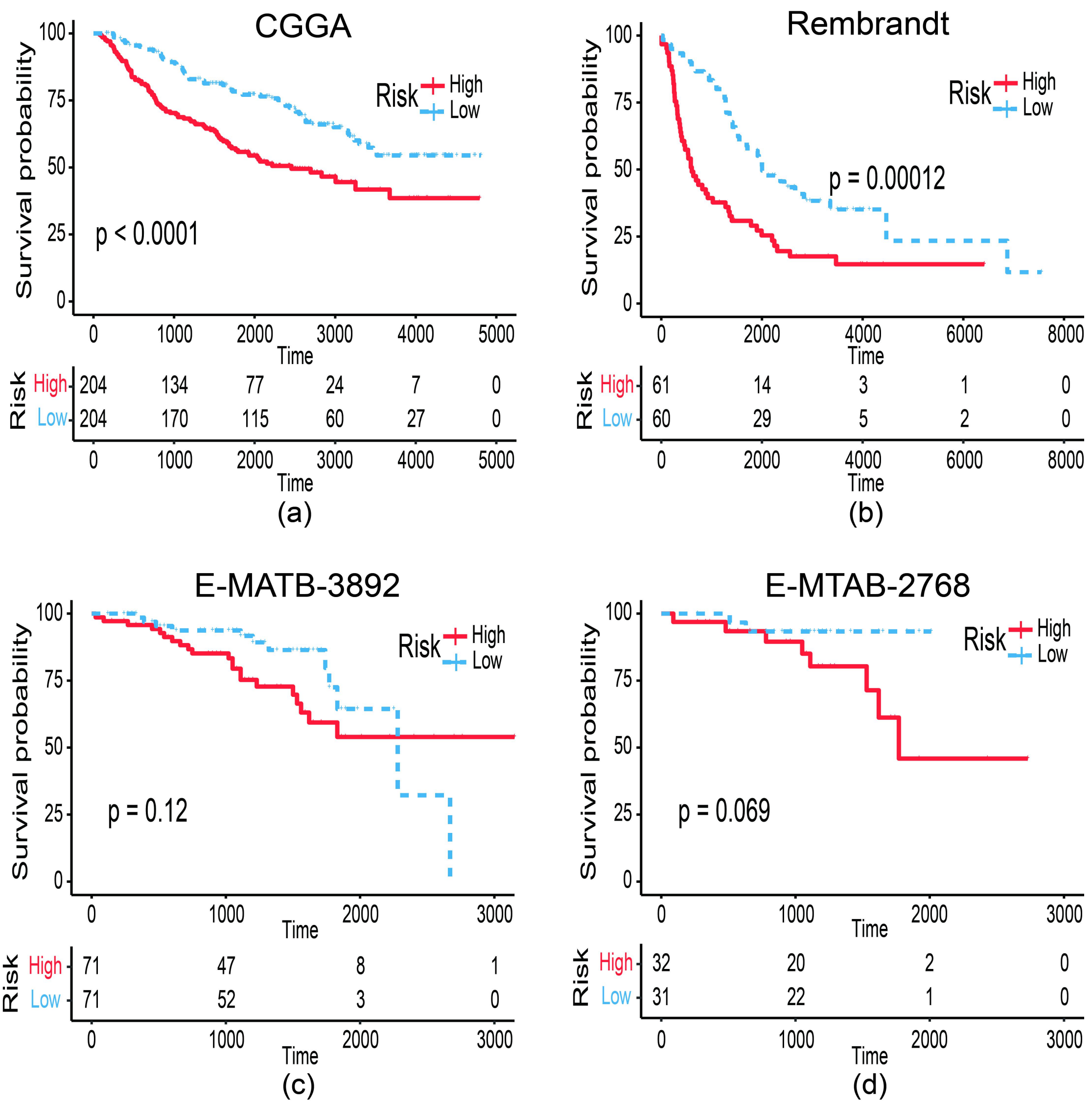

SUPPLEMENTARY FIGURE 9: Kaplan-Meier survival curves of OS between high-risk group and low-risk group using Zheng J (2022) in four external datasets. (a) Kaplan-Meier survival curves of OS between high-risk group and low-risk group in CGGA. (b) Kaplan-Meier survival curves of OS between high-risk group and low-risk group in Rembrandt. (c) Kaplan-Meier survival curves of OS between high-risk group and low-risk group in E-MATB-3892. (d) Kaplan-Meier survival curves of OS between high-risk group and low-risk group in E-MATB-2768.
